# Counselling Framework for Germline *BRCA1/2* and *PALB2* Carriers Considering Risk-Reducing Mastectomy

**DOI:** 10.3390/curroncol31010023

**Published:** 2024-01-09

**Authors:** Stephanie M. Wong, Carla Apostolova, Elisheva Eisenberg, William D. Foulkes

**Affiliations:** 1 Department of Surgery, McGill University, Montreal, QC H3G 1A4, Canada; 2 Stroll Cancer Prevention Centre, Sir Mortimer B. Davis Jewish General Hospital, Montreal, QC H3T 1E2, Canada; 3 Gerald Bronfman Department of Oncology, McGill University, Montreal, QC H4A 3T2, Canada; 4 Department of Human Genetics, McGill University, Montreal, QC H3A 0C7, Canada

**Keywords:** breast neoplasms, *BRCA1*, *BRCA2*, hereditary breast and ovarian cancer syndrome, mastectomy, cancer prevention

## Abstract

Female *BRCA1/2* and *PALB2* germline pathogenic variant carriers have an increased lifetime risk of breast cancer and may wish to consider risk-reducing mastectomy (RRM) for surgical prevention. Quantifying the residual lifetime risk and absolute benefit from RRM requires careful consideration of a patient’s age, pathogenic variant, and their personal history of breast or ovarian cancer. Historically, patients have been counselled that RRM does not necessarily prolong survival relative to high-risk surveillance, although recent studies suggest a possible survival benefit of RRM in *BRCA1* carriers. The uptake of RRM has increased dramatically over the last several decades yet varies according to sociodemographic factors and geographic region. The increased adoption of nipple-sparing mastectomy techniques, ability to avoid axillary staging, and availability of reconstructive options for most germline pathogenic variant carriers has helped to minimize the morbidity of RRM. Preoperative discussions should include evidence regarding postmastectomy sensation, the potential for supplemental surgery, pregnancy-related chest wall changes, and the need for continued clinical surveillance. Approaches that include sensation preservation and robotic nipple-sparing mastectomy are an area of evolving research that may be more widely adopted in the future.

## 1. Introduction

Given the elevated lifetime risk, many female *BRCA1/2* and *PALB2* germline pathogenic variant carriers (herein referred to as “carriers”) may wish to consider risk-reducing mastectomy (RRM) in order to prevent the development of breast cancer [1]. This article will summarize the current data on the perioperative considerations for RRM in women with high-penetrance breast cancer susceptibility genes, addressing the factors associated with optimal patient selection, surgical technique, and postoperative surveillance.

## 2. Risk Reduction with Mastectomy

### 2.1. Residual Lifetime Risk and Risk Reduction following Bilateral Mastectomy in Unaffected Carriers

**Figure 1:**
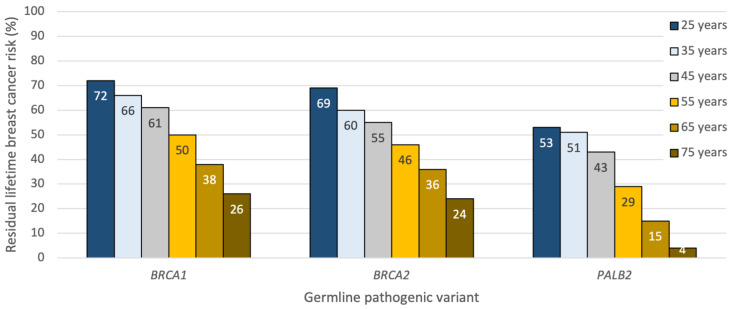
Approximate residual lifetime risk estimates for developing breast cancer in unaffected *BRCA1/2* and *PALB2* carriers by age and germline pathogenic variant. Derived from prospective cohort studies [2,3] and the ASK2ME clinical decision support tool [5].

The average woman with a germline pathogenic variant in *BRCA1/2* has a 69–72% (95% CI 61–79%) lifetime risk of developing breast cancer between the ages of 25 and 80 years old [2]. Pathogenic variants in the breast cancer susceptibility gene *PALB2* are associated with an approximate 53% (95% CI 44–63%) lifetime risk of developing breast cancer, increasing to 68% in those with multiple affected first-degree relatives [3]. For *BRCA1,* peak breast cancer incidence occurs between 41 and 50 years, whereas for *BRCA2* and *PALB2*, peak incidence occurs between 51 and 60 years of age [2,3]. As a result, patients considering RRM later in life will have already lived through a significant percentage of their cumulative risk and should be counselled accordingly; for example, a 70-year-old unaffected *BRCA1/2* or *PALB2* carrier harbors less than a 30% residual breast cancer risk to the age of 80 and may wish to consider enhanced surveillance over surgical prophylaxis. Thus, preventive decisions based on a patient’s “lifetime” risk should always incorporate a woman’s current age and her residual life expectancy [4] (Figure 1).

Early cohort studies evaluating the efficacy of bilateral total or subcutaneous mastectomy suggested a 90–95% relative risk reduction in *BRCA1/2* carriers electing for preventive breast surgery [6,7,8]. Subsequent prospective studies and a 2018 meta-analyses confirmed that the risk of breast cancer is dramatically lowered but not completely eliminated following bilateral RRM, with residual lifetime risk decreasing to approximately 1–2% [9,10,11,12]. The low residual risk of a subsequent breast cancer is attributed to the removal of, on average, 93% of the breast glandular tissue at the time of mastectomy [13,14]. In the prospective SKINI trial, investigators performed intraoperative biopsies of the skin envelope in ten to fourteen predefined locations following the removal of the breast. In 160 women undergoing therapeutic or RRM, residual breast tissue was detected in approximately half, with a median residual breast tissue of 7% per breast [14]. 

Real-world studies have since evaluated the incidence of breast cancer in unaffected *BRCA* carriers undergoing RRM versus surveillance. In 2019, an updated analysis from the *Hereditary Breast and Ovarian Cancer Netherlands* (HEBON) multicenter cohort reported an incidence of breast cancer in 0.7% of 1128 *BRCA1/2* patients who underwent bilateral RRM compared to 23.8% of 1729 carriers who elected for surveillance during a median follow-up of 10 years [15]. More breast cancers were detected in *BRCA1* carriers than in *BRCA2* patients (1% RRM vs. 27% surveillance in *BRCA1*; 0% RRM vs. 19% surveillance in *BRCA2*). Fortunately, the small number of patients who developed *BRCA1*-associated cancers in the post-RRM setting presented with small (<2 cm), node-negative tumors [15]. 

### 2.2. Ipsilateral and Contralateral Breast Cancer Risk in Affected Carriers with a History of Breast Cancer

Approximately 2.1–6% of women with breast cancer who undergo genetic testing will carry germline pathogenic variants in *BRCA1/2*, and 0.2–0.9% are carriers of a pathogenic variant in *PALB2* [16,17,18]. In affected carriers who have breast cancer, therapeutic mastectomy will prevent future primary cancers but does not mitigate the risk of an ipsilateral recurrence from one’s index malignancy. In one study of 114 therapeutic mastectomies performed in 105 *BRCA* carriers, 2.6% of patients developed a locoregional recurrence at a median follow-up of 5.8 years [19]. Another series of 325 *BRCA* carriers treated with mastectomy demonstrated a 5- and 10-year locoregional recurrence risk of 3.7% and 6.3% [20]. As in noncarriers, locoregional recurrence varies according to the tumor size and biologic subtype, nodal status, receipt of postmastectomy radiation, and use of/response to systemic therapy [21,22]. 

For affected carriers with a history of breast cancer, contralateral breast cancer risk varies according to the pathogenic variant and age at the time of the index breast cancer. In *BRCA1* patients, there is a 40% risk of developing contralateral breast cancer at 20 years, but this can range from as high as 60% to as low as 38% if under 40 or over 50 years old at the time of the first breast cancer diagnosis [2]. In *BRCA2* carriers, the 20-year contralateral breast cancer risk is 26% but also ranges from 20 to 68% depending on one’s age at the time of index breast cancer. In affected *PALB2* carriers who are premenopausal at diagnosis, a recent report found 10-year contralateral breast cancer risk to be 12.2%, increasing to 35.5% in those with ER disease [23]. In postmenopausal *PALB2* carriers, 10-year contralateral breast cancer incidence appears significantly lower at 5.1%. 

To mitigate the risk in known carriers, contralateral RRM can be considered and affords a similar risk reduction to unaffected carriers, with less than a 1–2% residual risk in the unaffected breast. As in unaffected patients, age and residual life expectancy should be included in counselling around contralateral RRM, and the stage and prognosis of one’s index breast cancer should also be factored into decision-making. 

### 2.3. Breast Cancer Risk in Affected Carriers with a History of Ovarian Cancer

In *BRCA1/2* carriers who have been treated for high-grade serous carcinoma of the ovary, fallopian tube, or primary peritoneum, the risk of developing a subsequent breast cancer is lower than reported for unaffected *BRCA* carriers. For these patients, overall survival is largely dictated by the ovarian cancer risk, as 10-year breast cancer incidence following an ovarian cancer diagnosis is less than 10% [24,25,26,27,28]. In one retrospective cohort of 184 *BRCA1/2* carriers with epithelial ovarian cancer, only 16 (8%) developed breast cancer at a median of 7 years’ follow-up [29]. Another multi-institutional cohort of 164 patients with *BRCA*-associated ovarian cancer demonstrated a 10-year breast cancer-free survival of 91%, with no deaths due to breast cancer [26]. Possible explanations for these findings include the putative preventive effect of platinum-based chemotherapy for ovarian cancer on the subsequent breast cancer incidence.

Given the low short-term breast cancer risk, RRM is not routinely considered in *BRCA* carriers with a recent history of ovarian cancer. However, overall survival following ovarian cancer typically plateaus beyond 8–10 years, after which RRM may be carefully considered on a case-by-case basis in ovarian cancer survivors [28,30]. The impact of 2–3 years of adjuvant PARP inhibitors on overall survival and subsequent breast cancer risk remains an area of ongoing investigation in this select patient population [31]. 

## 3. Survival following Mastectomy

**Figure 2:**
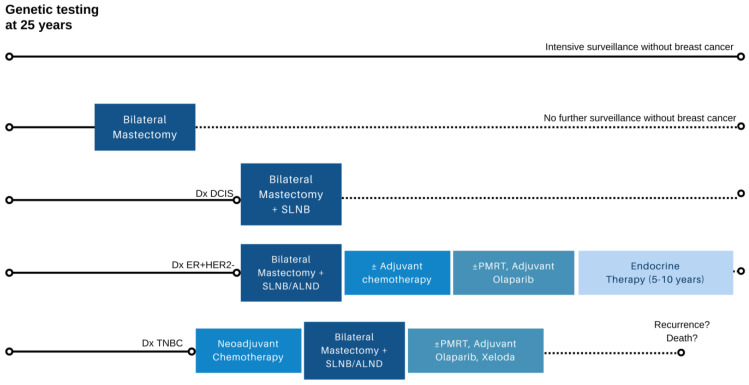
Possible breast-cancer-specific outcomes in *BRCA1/2* and *PALB2* carriers after genetic testing in early adulthood. Depending on specific germline pathogenic variant and family history, a significant minority of carriers who undergo surveillance may not develop breast cancer (row 1), while others may elect for bilateral risk-reducing mastectomy and forgo further imaging surveillance due to minimal residual lifetime risk (row 2). In carriers who develop breast cancer, treatment can vary depending on stage and tumor biology (rows 3–5), with the potential for recurrence and breast-cancer-related mortality. ***ALND*** axillary lymph node dissection; ***DCIS*** ductal carcinoma in situ; ***ER+HER2-*** estrogen receptor positive, HER2 negative breast cancer; ***PMRT*** postmastectomy radiation therapy; ***SLNB*** sentinel lymph node biopsy; ***TNBC*** triple negative breast cancer. Note: Axillary staging only required on the side of therapeutic mastectomy and if clinically indicated in the contralateral breast undergoing risk-reducing mastectomy.

Although RRM dramatically lowers the occurrence of breast cancer in *BRCA* carriers, to date, few studies have demonstrated a survival advantage from RRM [9,32,33]. Simulation models estimating life expectancy according to various management strategies have shown that early RRM combined with risk-reducing oophorectomy (RRSO) at age 40 maximizes survival in unaffected *BRCA1/2* carriers. However, RRSO with routine breast MRI and mammography offers relatively comparable outcomes, with a 2–3% decrement in survival by the age of 85 [34]. Models also highlight that as the age of RRM increases beyond 40 years, the estimated overall survival benefits diminish [34,35,36]. Thus, it is important for providers to reassure patients opting for surveillance that the avoidance of RRM will not dramatically alter their life expectancy. Instead, the primary aim of RRM is to reduce the risk of developing breast cancer and avoid the potential chemotherapy, endocrine therapy, and radiation therapy that accompany a breast cancer diagnosis [37] (Figure 2).

The results from population-based cohorts suggest that distinguishing between *BRCA1* and *BRCA2* carriers is highly relevant when considering survival in unaffected carriers opting for RRM. In the Dutch HEBON cohort, death from breast cancer was seen in 2% of *BRCA1* carriers electing for surveillance vs. 0.1% of *BRCA1* carriers electing for bilateral RRM (HR 0.06, 95% CI 0.01–0.46). In *BRCA2* carriers, there were no deaths from breast cancer in those undergoing RRM versus 0.9% in those electing for surveillance, precluding the calculation of hazard ratios [15]. The authors concluded that RRM was associated with a lower mortality in *BRCA1* carriers but not necessarily for *BRCA2* carriers. The characteristics of breast cancers that develop in *BRCA1* carriers, including higher rates of early onset, high-grade, and triple-negative disease, provide possible explanations in support of these real-world survival differences [38].

For carriers with a history of breast cancer, long-term data from retrospective studies have suggested a possible survival benefit associated with contralateral RRM, although analyses are likely to be subject to selection bias [39]. In 390 *BRCA1/2* carriers diagnosed between 1975 and 2009, the 20-year overall survival was 88% in those undergoing bilateral mastectomy versus 66% in patients undergoing unilateral mastectomy. However, the propensity score analysis of 79 pairs matched on BRCA mutation, tumor size, nodal status, age and year of diagnosis, and radiotherapy, tamoxifen, and chemotherapy receipt failed to show significant survival differences between the unilateral mastectomy and contralateral mastectomy groups (*p* = 0.08). The authors concluded that reductions in second primary contralateral breast cancers are likely to have a favorable impact on breast-cancer-specific mortality; however, given the small number of events in the cohort, further research was needed to confirm the findings [39]. 

## 4. Uptake of Risk-Reducing Mastectomy

The decision to undergo RRM in carriers is often highly personal, nuanced, and complex [40]. Age, marital status, parity, and a family history of breast cancer have all been shown to influence the decision to undergo RRM [41,42,43,44]. The pathogenic variant also plays a role, with *BRCA1* carriers more likely to elect for RRM over *BRCA2* carriers [42,45]. In those opting to undergo surgical risk reduction, the majority do so within 2–3 years following genetic testing, with the exception of women under 30 years [45]. In one cohort study of 887 unaffected carriers in the United Kingdom, almost all (88%) women who underwent bilateral RRM did so before the age of 50 years, although fewer than 10% were under the age of 30 [45]. In the literature, the average age of RRM in unaffected carriers ranges from 39 to 42 years old [45,46].

The uptake of RRM in *BRCA1/2* carriers has progressively increased since the early 2000s [47]. In a 2008 international study of 1383 unaffected *BRCA* carriers, Metcalfe et al. reported that 18% had undergone RRM, with significant geographic variation in uptake [48]. A 2019 follow-up study evaluating 6223 carriers demonstrated a 28% rate of RRM, although geographic variation remained equally pronounced [46], with the highest rates of RRM reported in the United States (approximately 50% of *BRCA* carriers) and the lowest rates in Poland, Israel, and South Korea (less than 5% of *BRCA* carriers) [43,46,49] (Table 1).

## 5. Skin- versus Nipple-Sparing Mastectomy

Skin-sparing mastectomy (SSM), a procedure that removes the nipple and areola complex with the underlying breast glandular tissue, was adopted as an oncologically safe option for *BRCA1/2* carriers undergoing RRM in the early 2000s [54]. Although initially controversial, the use of nipple-sparing mastectomy (NSM), which retains the nipple areola complex, increased in popularity in the late 2000s and steadily thereafter [55,56,57]. While keeping the nipple areola complex was initially felt to increase residual breast cancer risk, proponents of NSM cited improved cosmesis, patient satisfaction, and psychosocial well-being as important considerations driving the increased uptake [57,58,59,60]. At the time, surgeons also argued that the earliest cohorts of NSM incorporated women treated with “subcutaneous mastectomy”, an approach that included retention of up to 10 mm of subareolar tissue in order to maintain nipple viability [61]. Subsequent ex vivo anatomic studies demonstrated that it was feasible to safely remove nearly all ductal tissue underlying the nipple dermis while maintaining adequate perfusion [62,63]. With low rates of ischemic complications after the meticulous removal of subareolar tissue, NSM soon became more widely adopted for risk reduction in high-risk patient populations. 

Residual breast glandular tissue is known to be higher following NSM [64], although whether this translates into clinically meaningful differences in the subsequent breast cancer risk remains to be seen. In volumetric MRI analysis of 105 *BRCA1/2* patients, the proportion of total breast glandular tissue present within a 5 mm subareolar depth was only 1.3%, suggesting that nipple preservation adds minimal oncologic risk [65]. In the recent Swiss SKINI Trial, investigators prospectively evaluated the presence of residual breast tissue in 160 women undergoing mastectomies (32% for risk reduction). The trial showed that residual breast glandular tissue was present in 68.9% of NSM vs. 40.4% of SSM (*p* < 0.001) but did not quantify the median added volume of residual breast glandular tissue present [14]. Despite these findings, reassuring data on the oncologic safety of NSM have continued to emerge over the last decade. Multiple cohort studies of NSM have documented negligible rates of subsequent cancer directly involving the nipple or areola [27,66]. In a large, multicentered cohort study of 548 NSMs performed on 346 *BRCA1/2* carriers across nine institutions in the United States, no new breast cancers were diagnosed on the side of the procedure at a median follow-up of 34 months [67]. Studies with long-term follow-up are ongoing to assess whether this risk reduction is sustained beyond 5–10 years and whether similar results are seen in women with other high-penetrance pathogenic variants undergoing NSM, including *PALB2*, *TP53*, *PTEN*, and *CDH1* carriers.

## 6. Axillary Staging and Occult Breast Cancer at the Time of RRM

An occult invasive malignancy is detected in less than 1% of RRMs performed on *BRCA1/2* carriers with normal preoperative imaging. In one series by Kaas et al., only 1 (0.7%) of 147 unaffected carriers undergoing bilateral RRM had an invasive breast cancer diagnosed on final pathology, whereas 6 patients had ductal carcinoma in situ (DCIS) [68]. In our own series of 243 carriers (including those with *BRCA1/2* and *PALB2* but also *TP53*, *CDH1*, and *PTEN*), only 3.5% of mastectomies contained DCIS, while 0.8% contained invasive breast cancer [69]. Patient-level factors associated with occult malignancy included older age (over 60 years), prior breast cancer, BIRADS 4 findings on preoperative MRI, and the need for preoperative breast biopsy. As a result, we routinely perform MRI within 6 months of RRM to evaluate the need for axillary staging. In patients with BIRADS 1–3 findings on preoperative MRI, the risk of occult invasive malignancy remains less than 1%, allowing for the successful omission of sentinel lymph node biopsy in most women. In those with BIRADS 4 findings or the need for a preoperative biopsy, the risk of occult invasive breast cancer increases to 6–8%, prompting us to recommend sentinel lymph node biopsy regardless of the preoperative biopsy result [69]. Alternatively, one could consider the use of second-generation tracers containing superparamagnetic iron oxide nanoparticles that remain in axillary lymph nodes for 30 days after injection. The use of these agents allows for delayed sentinel lymph node biopsy to be performed as a second surgery, only if an occult invasive malignancy is found during the final pathology [70]. If clinically indicated (i.e., in these select women undergoing RRM with BIRADS 4 findings during preoperative MRI), surgeons should advise patients around the potential for skin staining and hyperpigmentation secondary to dye injection [71].

## 7. Reconstructive Options

Most women who undergo RRM elect to undergo reconstruction with either implant-based (alloplastic) or autologous flap-based approaches. Implant-based reconstruction can be performed in a two-stage fashion, with initial tissue expander placement, followed by exchange with a permanent implant or as a single-stage, “direct-to-implant” reconstruction at the time of mastectomy. Implants may be placed under the pectoralis major muscle (retropectoral or subpectoral), partially submuscular (dual-plane), or above the pectoralis muscle (prepectoral) [72]. Over the last decade, techniques in implant-based reconstruction have shifted from two-stage fully submuscular tissue expander (TE) placement to single-stage dual-plane or prepectoral reconstruction with or without an acellular dermal matrix [73]. The advantages of prepectoral reconstruction include reduced postoperative pain, the avoidance of animation deformity (superior implant migration with pectoral muscle contraction), and improved cosmetic outcomes due to a more natural breast projection and contour [74,75,76]. However, due to the proximity of the implant to the skin flap, inadequate flap perfusion or skin complications may compromise the reconstructive outcomes in prepectoral reconstruction, limiting its use for all patients [72]. Furthermore, fat grafting may be required as an adjunct to decrease visible rippling, augment mastectomy flap thickness, and improve the breast contour following implant-based reconstruction, particularly in prepectoral cases [77].

While over 90% of patients undergoing bilateral RRM elect for implant-based reconstruction [78], autologous flap-based approaches are gaining in popularity. Overall, autologous reconstruction accounts for 19% of all postmastectomy reconstructions performed in the United States. Since 2018, deep inferior epigastric perforator (DIEP) flaps have largely supplanted transverse rectus abdominis muscle (TRAM) and latissimus dorsi flaps as the autologous option of choice. This is largely due to lower rates of donor-site morbidity (hernia, bulge formation, pain) with DIEP flaps over traditional autologous approaches [79]. Historically, autologous reconstruction has been associated with longer operative times, increased resource utilization and length of stay, and prolonged recovery. However, recent studies have also shown improved quality of life and aesthetic satisfaction following autologous reconstruction, supporting its use in motivated patients [80]. For *BRCA* carriers contemplating pregnancy or RRSO following RRM, there are limited published data on autologous flaps and subsequent laparoscopic surgery or pregnancy [81]. 

For women with macromastia or significant breast ptosis who desire reconstruction but would prefer to avoid implant-based approaches or autologous procedures with tissue transfer, chest wall perforator flaps or the Goldilocks technique represent an attractive alternative. First described by Richardson and Ma in 2012, the Goldilocks reconstructive approach utilizes a Wise pattern reduction incision to reduce the size of the skin envelope, while the de-epithelialized skin and subcutaneous fat of the inferior mastectomy flap are used to reconstruct the breast mound [82]. The technique is safe for women with obesity or comorbidities who are poor candidates for conventional reconstruction and may allow for retention of the nipple on an inferior dermal pedicle [83,84]. As a reconstructive option, the Goldilocks mastectomy is associated with lower complications, reduced operative and recovery times, and an increased likelihood of avoiding postoperative drains. Moreover, it represents an ideal alternative to total mastectomy in developing countries where the cost of implants limits the reconstructive options [85]. 

## 8. Supplemental Surgery after Risk-Reducing Mastectomy

Supplemental surgeries and postoperative complications following RRM are common in *BRCA1/2* and *PALB2* carriers. The reoperation rates in women undergoing bilateral RRM vary widely in the available literature from 15.8 to 56.7% [86,87,88,89]. In a large cohort of *BRCA1/2* and *PALB2* carriers that examined supplemental surgeries, the rate of unanticipated reoperation at 5 years was 39.2%, with most women (72.3%) requiring a single additional surgery [89]. Women who experienced a postoperative complication in the first 30 days following surgery had a significantly higher risk of having two or more supplemental surgeries when compared to women without early postoperative complications (41.2% vs. 10.7%) [89]. Current studies report early postoperative complication rates between 13.2 and 28.6% [87,89].

Indications for supplemental surgery in carriers following RRM opting for implant-based reconstruction most commonly pertain to implant-related issues [86,88]. Nurudeen et al. performed a retrospective cohort study of 178 *BRCA1/2* carriers undergoing RRM with reconstruction and found that of the 136 women who had implant-based reconstruction, 27.8% required either an implant exchange or a capsulotomy [86]. In another large retrospective cohort study of 1099 women with a family history of breast cancer who underwent RRM with implant reconstruction, Zion et al. showed that 62% retain their original implants 20 years after placement [88]. The factors associated with a higher risk of supplemental surgery include older age, smoking, and parity [86,87,88]. In women who elect to undergo RRM without reconstruction, the rate of supplemental surgery is lower, ranging from 15.2 to 21% [88,89]. 

## 9. Sensation after Mastectomy

Sensation in the skin and nipple areola complex is often significantly attenuated after RRM. In most women, the medial and lateral cutaneous branches of the third to fifth intercostal nerves provide dominant sensation to the breast and nipple and are interrupted subcutaneously and/or at the level of the chest wall during surgery [90]. Many studies report considerable loss of sensation or total numbness following mastectomy [91,92], although recovery of some sensation after therapeutic mastectomy has been reported in 20–40% [60,93]. Unfortunately, because different modalities are often used to assess sensation postoperatively (self-reported questionnaires [60,91,93,94,95], Semmes Weinstein monofilaments [92,96], or paper contact [97]), a comparison of outcomes across studies with different follow-up times remains difficult. In one prospective study of 53 patients undergoing mastectomy with reconstruction, sensation at one year was present in only 20% of areas tested in SSM patients [92]. Interestingly, postoperative sensation was slightly better following NSM, with objective bilateral nipple sensation present in 28% of patients and up to two thirds of patients demonstrating the preservation of nipple sensation in at least one nipple [92].

Two European studies have specifically examined sensation in women undergoing RRM. A Swedish prospective study by Gahm et al. included 46 women undergoing bilateral RRM with immediate subpectoral implant-based reconstruction [98]. At a median of 2.4 years’ follow-up, they reported a retention of nipple sensation in 38% of breasts, although significantly higher touch thresholds were present compared to the baseline. Furthermore, cold and warmth thresholds were 8–9 degrees lower or higher, on average, when compared to the preoperative baseline. Notably, NSM was not associated with increased sensitivity of the nipple compared to patients who underwent SSM with free nipple grafts. Another study by van Verschuer et al. in the Netherlands analyzed 45 patients who underwent bilateral prophylactic NSM and SSM [96]. Using the validated BREAST-Q^®^ questionnaire, they showed lower sensitivity of the nipple areola complex when compared to the baseline in the NSM group.

## 10. Psychological Outcomes following Risk-Reducing Mastectomy 

Studies examining the long-term psychological impact of bilateral mastectomy in *BRCA1/2* carriers tend to show sustained reductions in anxiety and cancer-related distress following surgery [99,100,101]. Relative to age and risk-matched controls, patients report a lower perceived breast cancer risk after RRM [100]. In a recent prospective study of 98 *BRCA1/2* carriers in Germany, baseline anxiety levels were higher in women opting for RRM but decreased after surgery, whereas women opting for enhanced surveillance tended to have increased levels of anxiety over time [102]. A Dutch study of 96 *BRCA1/2* carriers using the standardized BREAST-Q^®^ questionnaire found that patients undergoing RRM reported lower physical well-being but otherwise similar mean satisfaction with their breasts, psychosocial well-being, and sexual well-being relative to those undergoing enhanced surveillance [103]. Although early studies showed detrimental or neutral impact on quality of life relative to the baseline in women post-RRM, more recent studies suggest that quality of life may even improve in up to 82% following RRM [104]. 

Although the majority of women undergoing RRM are satisfied with their choice and report low levels of regret, many acknowledge that coming to the decision to undergo surgery was stressful and emotional [105,106]. Physician-initiated discussions leading to bilateral RRM appear to be associated with higher levels of regret relative to discussions initiated by the patient themselves, suggesting that women who feel pressured into considering surgery may be at an increased risk of regretting their decision [107]. A study assessing informational needs that inquired about what women wished they knew prior to undergoing RRM found that many reported concerns related to reconstruction, the longevity of implants, and the look and feel of their future breasts [108]. Patients also reported a strong desire to see photographs that would allow them to prepare for the final result. Satisfaction with the information obtained preoperatively appears to be directly related to satisfaction with the postoperative outcome, highlighting the importance of informed, shared decision-making around RRM [109].

## 11. Postmastectomy Surveillance 

The current guidelines recommend continued annual chest wall examination after RRM, as the low residual risk does not appear to justify routine postmastectomy imaging. In one Israeli cohort study of 237 *BRCA1/2* carriers, Kanana et al. reported a surveillance protocol of physical examination with alternating ultrasound or MRI imaging every 6 months following RRM [110]. After 10-year surveillance, no breast cancers were diagnosed in 53 unaffected carriers who underwent bilateral RRM; however, 7% of 184 affected carriers developed a local recurrence. The authors noted that the locoregional recurrence rates were similar to noncarriers following mastectomy and highlighted that most recurrences were detectable using physical examination, although MRI detected tumors at smaller sizes. Another retrospective cohort study from the United States lends support for clinical examination as primary surveillance for patients post-RRM. In their cohort of 99 postoperative *BRCA1/2* unaffected carriers, two thirds of patients were followed with clinical examination alone, while the remaining had clinical examination with either baseline MRI (5%), annual MRI (4%), or MRI and/or ultrasound at irregular intervals (23%). At the 3-year follow-up, no new breast cancers had been diagnosed, while two patients underwent additional biopsies due to abnormalities detected on clinical breast examination alone [111]. 

## 12. Nipple Discharge and Pregnancy-Related Changes following Risk-Reducing Mastectomy

Nearly half of women who become pregnant after NSM report some clinical change in the reconstructed breast during the immediate pre- or postpartum period [112]. Benign nipple discharge may occur in pregnant patients following NSM and is felt to stem from residual microscopic glandular tissue present on the skin flaps. In one study of 27 patients (44% *BRCA1/2* carriers) aged 27 to 47 who became pregnant following NSM, 18.5% reported spontaneous milky nipple discharge from multiple ducts shortly after delivery [113]. The nipple discharge was small in volume and self-limited, resolving spontaneously within weeks to months. In contrast, the rate of nipple discharge in 1593 NSM patients without a postoperative pregnancy was only 0.25%, the majority of whom reported a spontaneous and watery discharge. The authors posited that the discharge likely resulted from persistently patent nipple duct orifices that permitted drainage of fluid from around the implant-based reconstruction.

## 13. Future Directions: Sensation-Sparing and Robotic Nipple-Sparing Mastectomy 

Sensation-sparing approaches continue to evolve with the primary aim of improving nipple areola complex sensation following mastectomy. Neurotization after autologous or implant-based reconstruction utilizes coaptation between a nerve graft placed between the third, fourth, or fifth intercostal nerve and the nerve that supplies the nipple areola complex [114]. Considerable literature to date has focused on neurotization after autologous breast reconstruction, showing positive results when measuring sensation compared to non-neurotized reconstructed breasts [115,116,117,118,119]. Two small series by Peled et al. and Djohan et al. comprised 16 and 13 patients, respectively, and focused on neurotization after implant-based reconstruction [120,121]. Peled et al. assessed nipple areola complex sensation using the two-point discrimination test and demonstrated preserved sensation in 87%, worsening in 9%, and improved sensation in 4% [121]. Two of the patients in the study by Djohan et al. underwent unilateral neurotization after bilateral reconstruction. When comparing the patients’ non-neurotized breasts to their contralateral neurotized breasts, they found that the latter had better sensation in six of the eight areas tested [120]. Two large reviews, assessing twenty-three studies each, looked at neurotization after both autologous and implant-based reconstruction [122,123]. These studies found promising results for neurotization after reconstruction, although both concluded that more research is required before neurotization can be adopted as the standard of care. At present, a lack of patient understanding and insurance coverage, coupled with hesitation on the part of surgeons due to additional time and cost, are felt to hinder the widespread implementation of neurotization in reconstruction procedures [124]. 

In parallel with sensation-sparing mastectomy, robotic or robot-assisted NSM was first introduced in 2015 and has gradually gained interest in the breast surgical community. A major advantage of robotic NSM is the smaller incisions required relative to conventional NSM; a multi- or single-port robotic platform allows for a 20–55 mm incision placed along the midaxillary line [125]. Following the creation of the workspace via initial open dissection within the standard subcutaneous plane, the robotic arm is docked, and the breast flaps retracted via CO_2_ insufflation. Following mastectomy, reconstruction proceeds without the robot, either with implants or DIEP flaps, utilizing the thoracodorsal artery and vein for microvascular anastomosis [126]. Early feasibility studies of robotic NSM showed a rapid reduction in operating room time after several cases, with surgeons requiring 20–30 robotic NSMs prior to learning curve stabilization [125,127]. In a recent Italian randomized controlled trial of 80 patients comparing robotic NSM to conventional NSM, Toesca et al. reported better patient-reported satisfaction and physical and sexual well-being with the use of robotic approaches [128]. Complication rates were similar between the two groups, and at a median follow-up of 42 months, there were also no differences in local recurrence, disease-free survival, or overall survival. Ongoing prospective cohort studies include the *Mastectomy with Reconstruction including Robotic Endoscopic Surgery* (MARRES) study and multicentered *Robot-assisted versus Open Nipple-sparing Mastectomy with Immediate Breast Reconstruction* randomized controlled trial. These plan to enroll nearly 3000 patients combined and will provide long-term, higher-level evidence around the use of this technology in high-risk patient populations undergoing RRM.

## 14. Conclusions

For patients considering RRM, weighing the benefits of surgery over enhanced surveillance requires careful consideration of the residual lifetime risk, specific pathogenic variant, and personal history of breast or ovarian cancer. Although recent cohort studies suggest a possible survival benefit for prophylactic mastectomy in unaffected *BRCA1* carriers, there are no data to suggest a survival benefit relative to MRI surveillance in unaffected *BRCA2/PALB2* carriers or carriers with a prior history of breast cancer. Despite the minimal survival advantage, the use of RRM continues to increase in germline pathogenic variant carriers, likely, in part, to the widespread adoption of NSM, expanded reconstructive options, and the ability to avoid axillary staging and radiologic surveillance postoperatively. Although further studies are warranted, sensation preservation and robotic NSM present promising future approaches that have the potential to further reduce morbidity and increase the uptake of risk-reducing surgery in appropriately selected patients.

## Figures and Tables

**Table 1:** Modern studies examining uptake of risk-reducing mastectomy in unaffected *BRCA1/2* carriers across varying geographic regions [15,43,45,46,49,50,51,52,53]. ***BRRM*** bilateral risk-reducing mastectomy.

Study	Country	No.	Overall % BRRM	*BRCA1* (% BRRM)	*BRCA2* (% BRRM)
Metcalfe et al. (2019) [46]	USA	774	49.9%	-	-
Heemskirk et al. (2019) [15]	Netherlands	2857	39.5%	42%	35%
Metcalfe et al. (2019) [46]	Canada	1005	38.0%	-	-
Marcinkute et al. (2022) [45]	UK (Manchester)	887	34.5%	37%	32%
Long et al. (2018) [50]	UK (Wales)	280	34.0%	-	-
Skytte et al. (2010) [50]	Denmark	206	30.0%	33%	25%
Petelin et al. (2019) [51]	Australia	493	27.6%	31%	25%
Singer et al. (2014) [51]	Austria	246	21.4%	-	-
Galmor et al. (2021) [43]	Israel	427	9.6%	12%	7%
Jung et al. (2020) [49]	South Korea	514	1.2%	-	-
